# The Report of the Lords Committee

**Published:** 1892-07-02

**Authors:** 


### The Hospital

An Institutional Journal of

Science, Medicine, Nursing, and Philanthropy.

*For Hospitals, Asylums, Infirmaries, Dispensaries, Medical Practitioners, Students, Nurses, and the Charitable Public.*

Vol. XII.—No. 301.] [July 2, 1892.

### The Report of the Lords' Committee.

The first conviction which a study of the Report of the Lords' Commission on Hospitals produces in the mind is that a vast mass of evidence has been carefully and intelligently collected, and that the conclusions arrived at and the suggestions offered are, for the most part, both reasonable in themselves and the natural outflow of the evidence. There are two ways open to us of dealing with the Report. We may look it through carefully and note its main points, or we may study it in detail and strive for the realisation of its suggestions. Intelligent and conscientious hospital managers will, we believe, do both these things. We protest strongly against the doctrine that the effect of an honest enquiry by competent investigators is to cease with the publication of their report. For our part we find the Report a storehouse of facts and arguments for the better management of all classes of hospitals, and it is our purpose to return to it from time to time with the object of their continuous improvement.

The Commissioners began at the beginning, and they did wisely. They have presented us with a brief history of all the most important London hospitals. People who never knew before may know now that there are three chief classes of medical charities in London, all of which go by the common name of "hospitals." There are first of all the great endowed hospitals, which are no more than three in number—namely, St. Bartholomew's, St. Thomas's, and Guy's. Next in order come the "Voluntary Hospitals." These constitute what, from the point of view of subscribers and philanthropists, may be called the "hospitals proper." They alone concern the people who give or withold subscriptions. The third class are the Poor Law or Workhouse Infirmaries. These are for the sole use of paupers, and are supported entirely by the rates. It is important to remember that the "Voluntary Hospitals," as they are called, are the only institutions which are supported by the contributions of the public, and it is these, and these alone, that newspaperwriters and philanthropists should keep within their purview when they are called upon to deal with questions of hospital management.

It is a pity that the Commission should have had to find serious fault with the management of the nursing department of St. Bartholomew's Hospital. St. Bartholomew's recently had an outbreak of diphtheria among its nurses and wardmaids. As many as twenty-three nurses and three wardmaids were attacked by the disease, and Dr. Thorne Thorne reported that the nursing home at this hospital was in a very unhealthy state, and that the drainage arrangements of the three principal ward blocks of the Hospital Square were defective and "could not be too strongly condemned." Concerning this the Commission report that the "neglect is, in the opinion of the Committee, the more inexcusable owing to the affluent circumstances of this charity." Now St. Bartholomew's is known to be a hospital and also a charitable institution; and it is commonly believed to belong to the class which depend for their support upon voluntary contributions. As a matter of fact, as we have pointed out, it is a richly endowed charity, which never appeals to the public for money at all, and whether its business be well or ill managed is no concern of hospital subscribers and ought not to influence their giving to other hospitals in the slightest degree.

The Commissioners have dealt with the "Voluntary Hospitals," or hospitals in the commonly understood sense of the word at great length. These include the general hospitals with medical schools, such as the London and Charing Cross, the general hospitals without medical schools, such as the Royal Free and the Metropolitan, and the vast mixed multitude of the special hospitals. The London Hospital in the Whitechapel-road is taken as the head and type of the voluntary hospitals proper. If space permitted we would here give an outline of the work done by the London Hospital for the eight hundred thousand industrious poor in whose locality it is placed. Such an outline would form an object lesson which would show at a glance the kind of services that all the voluntary hospitals of London are rendering, directly or indirectly, to every class of the community. For the present, however, we have to ask our uninitiated readers to take for granted or learn for themselves the extent and supreme excellence of the work done.

It was anticipated by many that the opening of the Lords' investigation would be the signal for an outbreak of adverse feeling against some or all of the hospitals. "Startling Revelations" were looked for, and "More Hospital Scandals" were confidently predicted. The London Hospital was selected to bear the brunt of the hostile attacks. It was quite an accident which determined the selection of the London. Any of the other voluntary institutions might as easily have been made the object of the onslaught. The Lords sifted the charges to the very bottom. Everybody was allowed to speak who chose; and, as a matter of fact, several did speak who seemed to know nothing whatever about the subject. It is impossible here to give more than the conclusions at which the Committee arrived; but these conclusions were, happily, quite decisive. In the words of the Report: "The charges are, on the whole, in the opinion of the Committee, not substantiated by the evidence. . . . In justice to the London Hospital, the Committee wish to add that it is an admirable hospital, doing work in a part of London where it confers inestimable benefits upon a very large and very poor population. They, therefore, think it is deserving of the greatest measure of charitable support." With the passing of the London Hospital through these searching fires, it was generally considered that the whole of the voluntary hospitals had been practically subjected to the same severe tests. The "London" emerged, not only unconsumed, but even unsinged; and all the hospitals may therefore be held to consist of the same pure gold.

The Commission had forced upon their attention a comparison, on the one hand between English and foreign hospitals, and on the other between the purely Voluntary Hospitals and the Poor Law Infirmaries, which are supported by the rates. On the general question of administration, the Committee find that the hospitals supported entirely by voluntary contributions bear a great resemblance to each other, and that "the evidence shows that they are generally well administered." They note "the enormous amount of work done by unpaid boards of managers; and the care exercised in the appointment of their medical as well as other officers." But by far the most important conclusion propounded by the Committee in this connection is, that the "Voluntary Hospital" system of London as a whole has no equal for efficiency and excellence of administration in any part of the world, and that the hospitals supported by voluntary contributions are to be preferred for many excellent reasons to the endowed hospitals on the one hand, and on the other to the rate-supported infirmaries.

The nursing question occupied many sittings of the Committee. We have not space to do more than summarise their conclusions. It is needless to state that the high level of efficiency to which nursing, as a skilled art, has attained was fully recognised. The Commission displayed a laudable anxiety for the safeguarding of the interests of nurses as well as for the protection of the public against the unskilled. They recommended, among other things, an eight hours working day; and they considered that every nurse in the large London hospitals should have an abundant supply of good food, not less than one full hour for dinner every day, at least two days off in the month, and three weeks holiday every year at some convenient period. These suggestions will commend themselves to all experienced and kindly hospital managers. The Commission considered that wherever it was possible pensions should be provided for nurses. In the words of the report, this should be done either "by the hospital following the example of the London and Guy's, by joining the National Pension Fund for Nurses, or by the hospital providing a special pension out of its own funds."

On the burning question of nurse registration the Report quotes with approval the opinion of Miss Nightingale and Mr. Rathbone, M.P., against the schemes of examination and registration proposed by the Royal British Nurses' Association. This question is discussed at great length, and the conclusion reached is that the idea of the protection of the public by the proposed methods of the Association is entirely fallacious. "If the Association," says the Report, "disclaims responsibility for the efficiency of the nurses whom it registers, it seems difficult to understand wherein lies the security which it offers to the public." We conclude as we began by expressing the conclusion that the Report of the Commission is a mine of suggestive wealth; and we hope our readers, as well as ourselves, will be disposed to devote serious study to its pages, and to graft its many reasonable conclusions upon the constitution and management of their hospitals.

